# Skeletal Stem Cells—A Paradigm Shift in the Field of Craniofacial Bone Tissue Engineering

**DOI:** 10.3389/fdmed.2020.596706

**Published:** 2020-11-23

**Authors:** Ruth Tevlin, Michael T. Longaker, Derrick C. Wan

**Affiliations:** 1 Division of Plastic and Reconstructive Surgery, Stanford University School of Medicine, Stanford, CA, United States,; 2 Hagey Laboratory for Pediatric Regenerative Medicine, Department of Surgery, Stanford University School of Medicine, Stanford, CA, United States,; 3 Institute for Stem Cell Biology and Regenerative Medicine, Stanford University School of Medicine, Stanford, CA, United States

**Keywords:** skeletal stem cells, skeletal progenitors, osteoprogenitors, craniofacial surgery, tissue regeneration

## Abstract

Defects of the craniofacial skeleton arise as a direct result of trauma, diseases, oncological resection, or congenital anomalies. Current treatment options are limited, highlighting the importance for developing new strategies to restore form, function, and aesthetics of missing or damaged bone in the face and the cranium. For optimal reconstruction, the goal is to replace “like with like.” With the inherent challenges of existing options, there is a clear need to develop alternative strategies to reconstruct the craniofacial skeleton. The success of mesenchymal stem cell-based approaches has been hampered by high heterogeneity of transplanted cell populations with inconsistent preclinical and clinical trial outcomes. Here, we discuss the novel characterization and isolation of mouse skeletal stem cell (SSC) populations and their response to injury, systemic disease, and how their re-activation *in vivo* can contribute to tissue regeneration. These studies led to the characterization of human SSCs which are able to self-renew, give rise to increasingly fate restricted progenitors, and differentiate into bone, cartilage, and bone marrow stroma, all on the clonal level *in vivo* without prior *in vitro* culture. SSCs hold great potential for implementation in craniofacial bone tissue engineering and regenerative medicine. As we begin to better understand the diversity and the nature of skeletal stem and progenitor cells, there is a tangible future whereby a subset of human adult SSCs can be readily purified from bone or activated *in situ* with broad potential applications in craniofacial tissue engineering.

## INTRODUCTION

Defects of the craniofacial skeleton may arise following trauma, diseases, oncological resection, or may be secondary to congenital anomalies. The craniofacial skeleton is an anatomically complex region with critical functional and cosmetic importance and thus, craniofacial reconstruction proves challenging. Calvarial defects impact protection of the central nervous system, meninges and brain whereas orbital defects can impact eye protection, support and protection. Maxillary and mandibular defects can impact the basic functions of speech, swallow and breathing in addition to cosmesis. Current treatment options are limited which highlights the importance of developing novel strategies to restore form, function, and aesthetics of missing or damaged bone in the face and the cranium. Reconstructive surgeons perform in excess of 50,000 craniofacial procedures annually to treat both congenital anomalies and head and neck defects (1), and the global bone graft and substitutes market size is expected to exceed $4 billion by 2026 (2).

To achieve optimal reconstruction, the goal is to replace “like with like” (3). Autologous tissue is preferred for reconstruction, but this approach can lead to significant donor site morbidity, may still result in resorption, and has limited availability (4, 5). As there is a finite supply of bone, alternative strategies have been pursued including cadaveric allografts tissue and alloplastic bone substitutes. Allografts are fraught with unpredictable rates of bone resorption and when processed to reduce immunogenicity, osteo-inductive factors are also deactivated. Alloplastic bone substitutes are complicated by infection, risk of extrusion, and an inability to grow with a developing child (6). With the inherent challenges of established options, there is a definitive need to develop alternative strategies to reconstruct craniofacial tissues.

Tissue engineering supports tissue regenerative processes by implementing cells, scaffolds, growth factors, gene manipulation, or combinations of these elements to reconstruct defects (5, 7, 8). Thus, by engineering and delivering tissues with or without cells capable of replacing damaged bone, regenerative medicine offers the potential to treat critical-sized bone defects which pose challenging clinical dilemmas. In the field of regenerative tissue engineering, there are multiple issues to consider in the creation of a functional, implantable replacement tissue. Importantly, there must exist an easily accessible, readily abundant cell source with the capacity to express the phenotype of the desired tissue and a biocompatible scaffold to deliver the cells to the skeletal defect (5).

Stem cells capable of skeletal regeneration are ideal cells for tissue engineering. Stem cells display tissue-specific differentiation patterns and have an ability to proliferate in response to certain physiological cues, critical to tissue regeneration (9). Adult stem cells have been isolated from multiple tissues including the central nervous system (10), skeletal muscle (11), adipose tissue (12), and from bone (13–17). Bones undergo myriad biologically important steps throughout their life cycle, such as morphogenesis and development, explosive growth and functional maturation, maintenance and repair of proper architecture and function, thus supporting the existence of adult skeletal stem cells (18, 19). There is a constant demand for differentiated cells at each sequential step so that bones can remodel, grow and become stronger, while maintaining their strength and function throughout life (19).

In this review, we will focus on the novel characterization of SSC populations with potential for implementation in craniofacial bone tissue engineering and regenerative medicine and describe their response to injury, systemic disease, and how their re-activation can contribute to tissue regeneration.

## MESENCHYMAL STEM CELLS

The most frequently studied cells for potential tissue regeneration are the mesenchymal stromal/stem cells (MSCs) which are reported to have the capacity to generate bone, cartilage, and fat, among other types of stromal cells (20). For decades, we and myriad others have investigated MSCs as an abundant pluripotent source for potential use in tissue engineering and regenerative medicine (21–23). MSCs have been reportedly isolated from the heart, liver, synovium, placenta, pancreas, cord blood (24), however the cells which encompass MSCs are heterogenous and their ability to differentiate into osteogenic progenitors occurs is not uniform (25). While preclinical trials using MSCs continue, a number of questions related to MSC characterization and their implementation are largely unresolved (18, 26). Although the mechanisms underlying the therapeutic effects of MSCs are not well-characterized in disease models, the search for consistent cell-surface markers to identify and harvest source-specific MSCs is paramount to improve patient outcomes. The success of MSC-based approaches has been hindered by heterogeneity of the transplanted cell populations which is mainly attributable to the lack of consistency in tissue source, but may also be a result of discrepancies in approaches to detection of a pure cellular population and isolation of prospective stem cells (18, 27).

## SKELETAL STEM CELL CHARACTERIZATION

**FIGURE 1 |:**
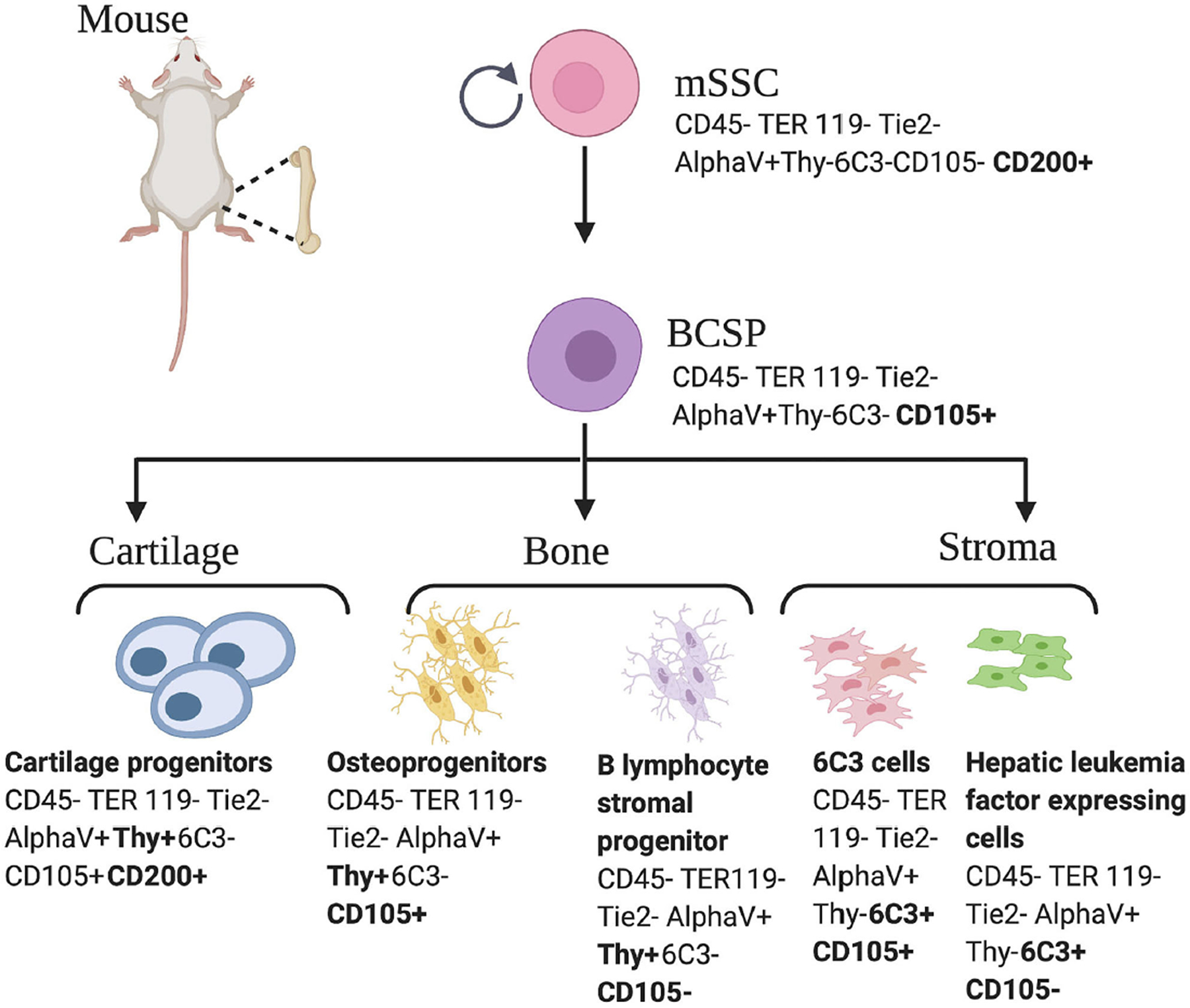
Mouse skeletal stem cell and downstream progenitors (13). Skeletal stem cells (SSCs) and their progenitors can be isolated from mice bones on the basis of distinctive immunophenotypes using flow cytometry. The mSSC is shown at the apex of the stem cell tree with differentiation into increasing fate restricted progenitors. The multipotent bone cartilage and stromal progenitor (BCSP) gives rise to mouse cartilage progenitors, mouse osteoprogenitors, mouse B lymphocyte stromal progenitors, mouse 6C3 stromal cells, and mouse hepatic leukemia factor expressing cells. The immunophenotype of the cell surface markers are shown for each cell (13, 34). This figure is adapted from Chan et al. (13).

The terms skeletal stem cells (SSCs) and MSCs are not equivalent and thus, should not be used interchangeably, as they report different skeletogenic populations (18). SSCs are distinguished by the local restriction of cells to bone with the ability to form an environment for hematopoiesis, the ability to self-renew on the clonal level, and the ability to demonstrate multipotency under *in vivo* conditions. Pioneering studies led by Friedenstein et al. established the presence of colony forming skeletogenic cells in the 1980’s (28). In the past decade, significant progress has been made in the characterization of cells capable of giving rise to bone, cartilage, and bone marrow stroma, hereafter referred to as skeletal stem cells (15, 16, 29–32). In 2015, following a combination of rigorous single cell analyses and lineage tracing technologies, our laboratory characterized the mouse skeletal stem cell (mSSC), a single cell capable of giving rise to bone, cartilage, and bone marrow stroma in mice (13). The mSSC immunophenotype is characterized by differential expression of AlphaV, Thy, 6C3, CD 15, and CD 200 (13). These cells are distinct from the MSC in that they do not give rise to adipocytes, fibroblasts, hematopoietic, or muscle cells (33). Furthermore, mSSCs defined a lineage tree of skeletal stem and increasingly fate-restricted progeny, which had differential ability to generate bone, cartilage, and bone marrow stroma (Figure 1). In addition, pathways were defined that play a role in directing the differentiation of skeletal stem and progenitor cells. Specifically, to address a large unmet clinical need, osteogenesis and chondrogenesis could be directed from mSSCs through manipulation of local niche signaling (13).

Building upon these findings, Worthley et al. demonstrated that the expression of a bone morphogenetic protein (BMP) antagonist, Gremlin 1, defines a specific population of SSCs in the mouse bone marrow (15). The so-called “osteochondroreticular” (OCR) cells self-renew, generate osteoblasts, chondrocytes, and bone marrow stromal cells, but do not give rise to adipocytes. These OCR stem cells are concentrated within the metaphysis of long bones and are implicated in bone development, remodeling, and fracture repair. OCR cells show great theoretical promise for skeletal tissue engineering, as these cells have been harvested from a donor animal, expanded *in vitro*, and transplanted both directly and serially into the femora of fractured recipient animals, resulting in osteochondral differentiation in the callus (15). Mizuhashi et al. also characterized a population of PTHrP+ chondrocytes in mice that possessed stem cell characteristics with overlapping immunophenotype to the mSSC as characterized by Chan et al. (13) and Mizuhashi et al. (35). On comparison of the mSSC to the mouse hematopoietic stem cell (HSC) tree, it is reasonable to deduce that mSSCs defined by varying surface markers and lineage tracing techniques reported by different groups may indeed overlap and/or fit together in a more complex lineage tree, similar to that of the HSC. As many studies confirmed that specific adult SSCs exist in the mouse, and given the conservation of skeletal genes in mice and humans (36), the search for human SSCs continued.

**FIGURE 2 |:**
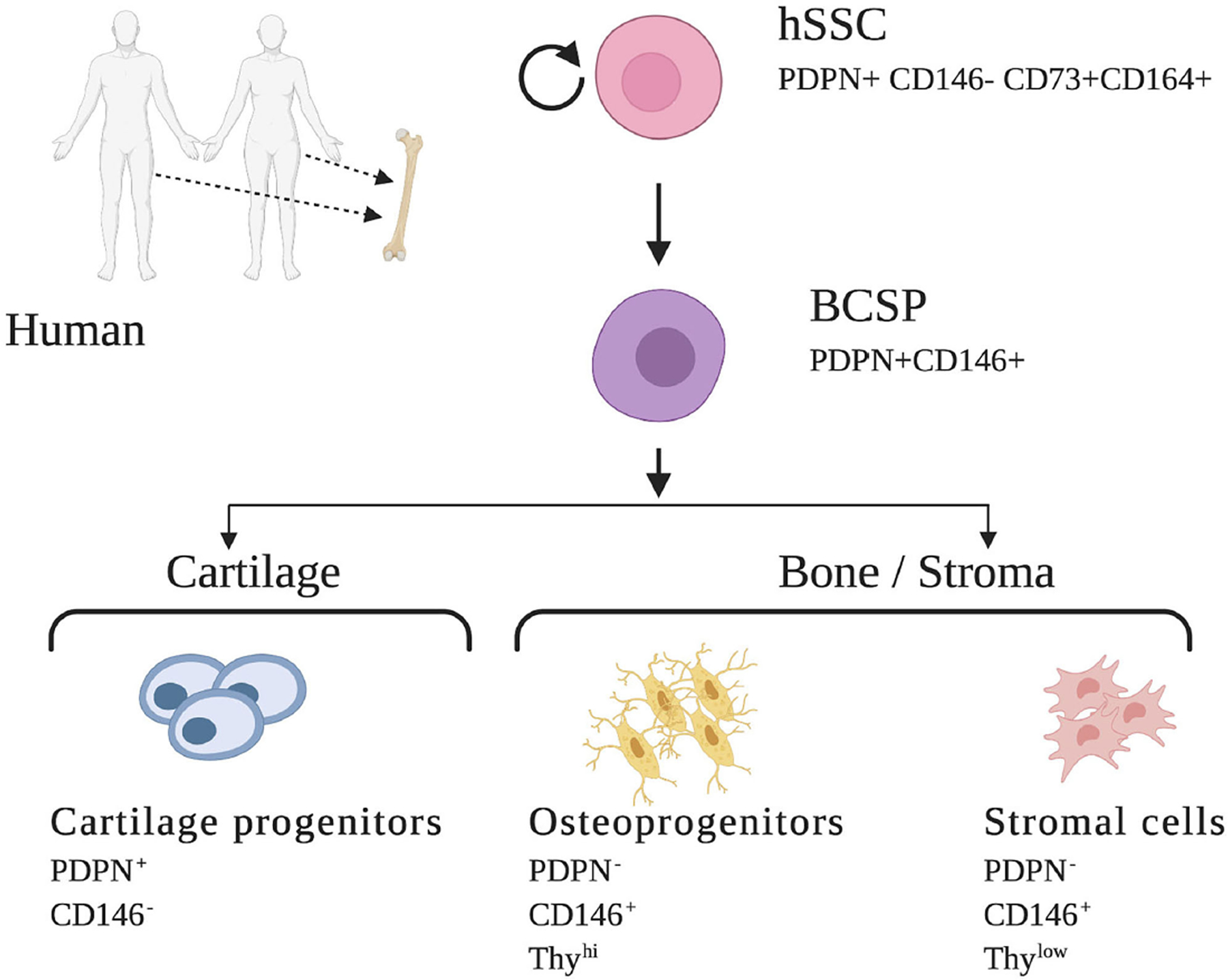
Human skeletal stem cell and downstream progenitors (14). Skeletal stem cells (SSCs) and their progenitors can be isolated from human bones on the basis of distinctive immunophenotypes using flow cytometry. The hSSC is shown at the apex of the stem cell tree with differentiation into increasing fate restricted progenitors. The multipotent bone cartilage and stromal progenitor (BCSP) gives rise to cartilage progenitors, osteoprogenitors, and stromal cells. The immunophenotype of the cell surface markers are shown for each cell (14, 34). This figure is adapted from Chan et al. (14).

Early efforts to isolate non-HSCs in human bone relied on the ability of bone marrow cells to adhere to plastic plates. However, these cultures of MSCs contained heterogenous mixtures of cells with indeterminate potential and inconsistent contribution to many overlapping lineages such as bone, cartilage, fat, muscle, fibroblast, endothelial cells, and stroma. Likely, these cells represent a population comprised of multiple distinct stem cells rather than a uniform purified skeletal stem cell (14). Identification of several specific cell-surface proteins led to enrichment for skeletogenic activity in MSCs and ultimately resulted in the identification of osteogenic, chondrogenic, and adipogenic lineages (14, 37). Building upon these findings and following characterization of the mSSC, significant progress was made to identify a homogenous human SSC population and its lineally related downstream progenitors. Using a set of mSSC-specific genes and their human orthologs in combination with rigorous single cell analyses, FACS isolation, and *in vivo* differentiation assays, Chan et al. were enabled to define an immunophenotype that characterized the human skeletal stem and increasingly fate-restricted progenitor cells characterized by differential expression of PDPN, CD146, CD 73, and CD164 (Figure 2) (14).

## SKELETAL STEM AND PROGENITOR CELL RESPONSE TO INJURY

Stem and progenitor cells mediate homeostasis and regeneration in postnatal tissue and alterations in tissue microenvironment are known to influence stem cell behavior. A true SSC should respond to injury, and previous studies report that bone injury induces progenitor expansion (32, 38). Skeletal fracture may thus activate a distinct subset of skeletal stem and progenitor cells in mice that mediate tissue regeneration and repair (39). Worthley et al. have described OCR SSCs to be involved in fracture repair (15). However, as endochondral bone formation has been found to occur through the bone cartilage and stromal progenitor (BCSP), which is derived from the SSC (13), these cells may also participate in long bone fracture healing. Using a model of transverse, mid-diaphyseal femoral fractures with intramedullary fixation, progenitor expansion was observed to precede ossified callus formation at multiple time points (39).

Murphy et al. also identified that mSSCs could be re-activated in the setting of a microfracture bone injury (40). Marecic et al. further identified injury induced phenotypic changes in the BCSP progenitor cells. Notably, the progenitor cells harvested from fracture calluses had significantly increased plating efficiency, as determined by colony number, significantly greater viability, and markedly reduced apoptotic activity. In addition, fracture-induced BCSPs had greater osteogenic potential when assayed both *in vitro* and *in vivo* in a heterotopic kidney capsule transplantation model. Using transcriptional and translational analyses, a highly potent regenerative cell type, the fracture-BCSP (f-BCSP), was thus identified that recapitulated many gene expression patterns involved in perinatal skeletogenesis (39).

Building on this work in human bone, hSSCs have also been found to be amplified in soft callus fracture specimens when compared to their frequency in uninjured skeletal tissues that were obtained from patients undergoing bone graft procedures (14). To further examine the activation of hSSC in response to injury, our group has established a new human bone xenograft mouse model to evaluate the effects of skeletal injury in human limb bones in a more controlled setting. Following transplantation and engraftment of human fetal phalangeal grafts (obtained from 18-weeks-old fetuses) into the flanks of 5-days old immunodeficient mice, uni-cortical fractures were induced on the xenografted phalangeal bones. This resulted in significant localized expansion of hSSCs relative to unfractured areas in the same bone. Therefore, consistent fracture induced expansion of both mSSC and hSSC in response to injury has been observed (14, 39), which has also been replicated in other SSC populations (15, 41).

## SKELETAL STEM AND PROGENITOR CELL NICHE RESPONSE TO SYSTEMIC CONDITIONS

Schofield first proposed the concept of a stem cell “niche” where stem cells are supported by a specialized micro-environment within tissues which promote their long-term stem cell activity (15, 42). Niches are able to balance the production of stem and progenitor cells to maintain tissue equilibrium (43) and niche activity is carefully regulated to ensure appropriate stem cell function (42, 44, 45). Tissue injury demonstrates the dynamic relationship between stem cells and their niches. A decline in niche function and the inability to guide local self-renewal contribute to reduced tissue homeostasis and repair in a number of systems (15, 46). In addition, changes in circulating systemic factors have also led to decreased stem cell activity (42, 47–52).

It has been proposed that interactions between SSCs and their downstream progeny act to maintain an appropriate pool of SSCs through paracrine signaling (53). In a mouse model of diabetes mellitus, high serum concentrations of tumor necrosis factor alpha has been shown to directly repress the expression of Indian hedgehog (Ihh) in mSSCs and in their downstream progenitors, leading to deficient niche signaling and impaired SSC bone regeneration (54). Furthermore, conservation of repressed Ihh signaling in human skeletal progenitors obtained from freshly dissected femoral and knee specimens in osteoarthritic diabetic patients undergoing total joint arthroplasty has been appreciated (54). In mouse models of obesity, ectopic adipocyte accumulation in the bone marrow is believed to contribute to age-related impairment of bone regeneration (55–57).

Heterotopic ossification (HO), defined by the formation of extra-skeletal bone which occurs in patients with substantial trauma or BMP type 1 receptor mutations (58, 59), likewise highlights the interplay between progenitor cells and their associated niche in the pathogenesis of disease. Agarwal et al. examined if known mouse osteoprogenitor cells which contribute to normal bone development are involved in HO (60). Importantly, BCSPs were found to be enriched at sites of HO, participating in *de novo* bone formation both in a trauma-induced mouse model for HO and in a transgenic mouse expressing a constitutively active type I Activin A receptor involved in BMP signaling leading to increased HO susceptibility. They further demonstrated that the BCSPs in HO undergo osteogenic and chrondrogenic differentiation on the basis of osteocalcin, osterix and sox9 expression. These results further support that BCSPs play a conserved role in post-natal bone formation given their shared presence in normally developing bone, healing fracture callus, and in *de novo* ectopic bone formation (60).

The capacity for skeletal tissue repair and regeneration declines with aging and thus bone repair is deficient in aging patients. Josephson et al. recently delineated that age-associated inflammation (“inflamm-aging”) is the main culprit for skeletal stem and progenitor cell dysfunction seen with advancing age (47). In contrast to the organized inflammatory response which follows trauma, chronic pro-inflammatory cytokines inhibit regeneration in a variety of tissues including bone (61). Josephson et al. identified that NF-κβ plays a central role in inflamm-aging and that modification of the inflammatory niche represents a valid translational approach to functionally rejuvenate aged skeletal stem and progenitor cells. By using a pharmacological approach inhibiting NF-κβ activation (sodium salicyclate), they demonstrated a functional rejuvenation of aged skeletal stem and progenitor cells with decreased senescence, increased stem and progenitor cell proliferation and increased osteogenesis (61).

Ambrosi et al. analyzed and isolated hSSCs from callus tissue of 61 patients ranging from 13 to 94 years for functional and molecular studies (62). These studies revealed that advanced age significantly correlated with reduced osteogenic and chondrogenic potential. Their results suggest that geriatric hSSCs preferably acquire a fibrogenic fate, which leads to deficient healing. Transcriptomic comparisons revealed downregulation of skeletogenic pathways such as Wnt and upregulation of senescence-related pathways in older vs. young hSSCs (62). Sirt1 has been proposed to increase stress resistance and cell death protection expression and was downregulated in geriatric hSSCs. As Sirt1 functions as a histone deacetylase, functional differences in hSSC may be epigenetically regulated during aging. Previous studies have shown increased lifespan and delayed aging by preserving Sirt 1 expression (63–66). Consequences of hSSC aging may be reversible as agonists of the Sirt1 histone deacetylase significantly improved osteogenic differentiation of aged-impaired hSSCs (62).

Aging may also have a detrimental impact on bone by acting on the hematopoietic niche. Aged bone stroma can be affected by age-related disruption of the hematopoietic niche where myeloid differentiation is dominant during hematopoietic stem and progenitor cell expansion (18). The age related change in niche signaling has a resultant downstream effect of increased osteoclastogenesis in the setting of diminished skeletal stem and progenitor cell pool, thus further reducing skeletal bone mass (18). Taken together, in aging there are changes in local niche signaling that lead to skewing of skeletal lineages to a fibroblastic phenotype, increased osteoclastogenesis and pro-inflammatory cytokine production which reduce bone regeneration (18), reflecting the importance of the niche on skeletal homeostasis and repair/regeneration.

## SKELETAL STEM CELL RESPONSE TO RADIATION

Osteonecrosis of the jaw can lead to significant loss of bone, loss of teeth and aesthetic deformity, resulting in significant disability and reduced quality of life (67). It is most commonly associated with radiation, when the radiation dose exceeds 50 Gy (68). Radiation, an important modality for treatment of many malignancies, has been shown to result in disorganization and coarsening of the stromal trabecular architecture (69), and this is also associated with a time-dependent loss of skeletal progenitors (70), limiting healing, and impairing normal homeostasis. Furthermore, this bone dysfunction has been found to correlate with reduced BCSP frequency in a mouse model of hind limb radiation prior to fracture injury. Reduced callus formation, prolonged healing, and significantly reduced BCSP osteoprogenitor expansion in the setting of prior radiation have all been observed (39), punctuating the important interplay between skeletal progenitors and the stromal niche. Sclerostin, a Wnt antagonist, has been recently proposed as a new target to reduce radiation induced osteopenia (71). Sclerostin knockout mice are insensitive to radiation (72–74). Chandra et al. hypothesized that neutralization of circulating sclerostin with a monoclonal antibody, leading to activation of the canonical Wnt signaling in bone, could treat osteoporosis by enhancing Wnt/β-catenin signaling and resulting in bone protection (71). The effect of Sclerostin and relevant methods of antagonism on skeletal stem cells and their progenitors offer an interesting focus of further investigation to aid patients undergoing radiation of the craniofacial skeleton.

## SKELETAL STEM CELL RE-ACTIVATION IN ENDOGENOUS MODELS

During regenerative processes, adult stem cell populations change not only in proliferation and location but also in their underlying gene-regulatory programs (75, 76), and in response to injury, stem cells may reactivate a greater potential for differentiation (77). Distraction osteogenesis (DO), an endogenous form of bone tissue engineering, has been used clinically to generate new bone. It was initially developed by Ilizarov for lengthening long bones after a chance discovery following a patient’s misunderstanding of post-operative instructions (78). Following placement of an external fixator, rather than compressing a bone fracture along its long axis, gradual guided separation of osteogenic fronts was found to result in *de novo* bone growth across the gap (78, 79). In DO, a surgical fracture (osteotomy) is created and subsequent expansion along its long axis is performed which creates mechanical stimulation, inducing biologic responses promoting bone regeneration. DO is divided into three key steps: a latency period for callus development, a distraction period of gradual lengthening, and a consolidation period that allows the stretched callus to mineralize. Distraction forces have been found to promote callus reorganization with a fibrous center, a highly vascularized zone of developing bone, and a mineralizing zone of bone undergoing primary bone formation adjacent to the uncut host bone. When tension is applied to move the bony segments apart, mineralization occurs from the bony ends toward the fibrous center.

The use of DO in the mandible has increased over the past two decades to treat severe bone deficiency, as occurs in Pierre Robin Sequence (PRS) or other craniofacial syndromes associated with micrognathia (79, 80). Historically children with PRS required tracheostomies, but the implementation of DO has obviated this need to address the compromised airway in newborns with low operative morbidity and improved QOL (81). DO has also been used in distraction of the midface (maxillary deficiency and craniofacial synostosis), zygoma (Treacher Collins syndrome), maldevelopment of the cranial vault (craniofacial synostosis), and edentulous mandible and maxillary alveoli (79, 81). Importantly, major bony structures of the face, including the maxilla and mandible, are derived from neural crest, and re-activation of neural crest transcriptional programs may be important for repair and regeneration of these skeletal elements (82, 83). In a mouse model of mandibular DO, Ransom et al. demonstrated that adult mSSCs revert to a developmentally plastic state, activating an embryonic neural crest-like transcriptional pattern (84). This was primarily driven through mechanical stimulation of focal adhesion kinase-mediated signaling pathways. Of note, the bone regenerated following DO may be associated with complications such as relapse, whereby the final length of the bone diminishes after the consolidation period. With a better understanding of the underlying biomechanics of mandibular osteogenesis and regulatory cues guiding SSCs, innovative clinical solutions in craniofacial skeletal regeneration can be developed.

The craniofacial sutures are a unique skeletal structure which continue to pose complex clinical dilemma in the setting of craniosynostosis, a condition which results from pathologic premature fusion of the cranial sutures. It has long been postulated that the suture mesenchyme is the niche of the SSCs essential for cranial morphogenesis. Recently an Axin-2 expressing stem cell population has been isolated from sutures and has been shown to contribute directly to calvarial defect repair and skeletal regeneration in a cell autonomous fashion (85). The only other marker to date capable of marking the cell population contributing to calvarial maintenance and injury repair is Gli1 (86). Maruyama et al. demonstrated that Axin 2 and Gli1 are co-expressed in a putative suture stem cell population which is predominantly found in calvarial bones, rather than bones of the axial skeleton and which is activated in the setting of calvarial injury (85, 86). In addition, Debnath et al. characterized the “periosteal stem cell,” which they isolated from long bones and calvaria of mice (87). The PSC displays clonal multipotency and self-renewal and is activated in the setting of long bone injury, highlighting a key characteristic shared by other SSC populations (87). Further studies of clinical samples harvested from patients with craniosynostosis also suggest that Axin 2 may reasonably represent a marker for the stem cell population that undergoes depletion during premature ossification process occurring in craniosynostosis (88). While this work is still in its infancy, it suggests that SSC activation may indeed be detrimental in this clinical setting, contributing to the development of craniosynostosis (85, 86, 88).

Another complex area for craniofacial tissue engineering is the temporomandibular joint (TMJ) which is imperative for daily functions such as talking and eating. The TMJ is a complex joint formed between the temporal bone and the mandibular condyle that can be affected by inflammatory and degenerative arthritis and can affect 8–16% of the population (89–91). In the advanced stage, surgical interventions such as condylotomy and arthroplasty are recommended but in earlier stages, palliative therapies are pursued rather than restorative therapies. Recently, Murphy et al. demonstrated that aging is associated with a progressive loss of SSCs and diminished chondrogenesis in the joints of both humans and mice (40). They activated a regenerative response by performing microfracture surgery where the surgeon drills through denuded cartilage into the marrow cavity. They reported that microfracture surgery alone tended to form fibrous tissue but with localized co-delivery of BMP2 and soluble VEGF receptor antagonist in addition to microfracture, they were able to skew the resident stem cell differentiation toward articular cartilage (40). These data suggest that microfracture along with local delivery of BMP2 and soluble VEGF antagonism could hold promise for patients who suffer with TMJ arthritis and induce *de novo* articular cartilage formation (40).

## LOOKING TO THE FUTURE OF CRANIOFACIAL TISSUE ENGINEERING

Mouse studies continue to power our understanding of skeletal development and regeneration. However, the characterization of human SSCs that have been identified in fetal tissue, adult fracture callus, and in xenograft models of injury holds enormous promise for a paradigm shift in the treatment of craniofacial defects and the understanding of pathogenetic mechanisms. *In vitro* methods to derive tissue specific stem cells, expand patient derived stem cells, and protocols to differentiate them into functional tissues are highly important topics in stem cell research and bioengineering. However, as has been seen with bone marrow derived MSCs (92), *in vitro* cell culture experiments are flawed with regard to the assessment of *in vivo* cell behavior and should not be considered as evidence of stem cell activity (18). SSCs should be able to self-renew, give rise to increasingly fate restricted progenitors, and differentiate into osteoblasts/osteocytes and chondrocytes, all on the clonal level *in vivo* without prior *in vitro* culture (24). We are only beginning to understand the diversity and the nature of skeletal stem and progenitor cells and how they actually behave *in vivo*. The discovery of a subset of human adult SSCs that can be readily purified from bone has broad potential applications in regenerative medicine. The hSSC exhibits all of the hallmarks of an early skeletal stem cell that can give rise to downstream lineage committed progenitors that may be beneficial in skeletal replacement therapies. Further studies, aiming to understand the mechanisms by which SSCs interact with the environment, interact with downstream progenitors and regulate their lineage choice under disease conditions are likely to unlock the regenerative potential of these cells and open up further therapeutic avenues for craniofacial skeletal tissue engineering.
